# Evaluation of General Practice Pharmacists: Study Protocol to Assess Interprofessional Collaboration and Team Effectiveness

**DOI:** 10.3390/ijerph18030966

**Published:** 2021-01-22

**Authors:** Thilini Sudeshika, Mark Naunton, Gregory M. Peterson, Louise S. Deeks, Jackson Thomas, Sam Kosari

**Affiliations:** 1 Discipline of Pharmacy, Faculty of Health, University of Canberra, Bruce ACT 2617, Australia; Mark.Naunton@canberra.edu.au (M.N.); g.peterson@utas.edu.au (G.M.P.); louise.deeks@canberra.edu.au (L.S.D.); jackson.thomas@canberra.edu.au (J.T.); sam.kosari@canberra.edu.au (S.K.); 2 Department of Pharmacy, Faculty of Allied Health Sciences, University of Peradeniya, Peradeniya 20400, Sri Lanka; 3 School of Pharmacy and Pharmacology, University of Tasmania, Hobart TAS 7005, Australia

**Keywords:** interprofessional collaboration, general practice, general practice pharmacists, team effectiveness, inclusion, evaluation

## Abstract

The inclusion of pharmacists into general practices has expanded in Australia. However, there is a paucity of research examining interprofessional collaboration and team effectiveness after including a pharmacist into the general practice team in primary or community care. This is a protocol for a cross-national comparative mixed-methods study to (i) investigate interprofessional collaboration and team effectiveness within the general practice team after employing pharmacists in general practices in the Australian Capital Territory (ACT) and (ii) to compare interprofessional collaboration and team effectiveness of pharmacists in general practice across Australia with international sites. The first objective will be addressed through a multiphase sequential explanatory mixed-method design, using surveys and semi-structured interviews. The study will recruit general practice pharmacists, general practitioners, and other health professionals from eight general practices in the ACT. Quantitative and qualitative results will be merged during interpretation to provide complementary perspectives of interprofessional collaboration. Secondly, a quantitative descriptive design will compare findings on interprofessional collaboration (professional interactions, relationship initiation, exchange characteristics, and commitment to collaboration) and team effectiveness of general practice pharmacists in Australia with international sites from Canada and the United Kingdom. The results of the study will be used to provide recommendations on how to best implement the role of general practice pharmacists across Australia.

## 1. Introduction

In Australia, general practice clinics are often the frontline primary health care service, with almost 90% of the population visiting a general medical practitioner at least once each year [1]. There is expected to be a 37.5% increase in the demand for general practice services between 2019 and 2030 [2]. The main factors driving this demand are the ageing population and the increasing impact of chronic diseases [2,3,4,5]. Higher demands for general practice services can cause excessive workload and stress for general practitioners, which may lead to lower quality of patient care [6,7]. The allocation of appropriate service provision to other primary health-care professionals can assist in addressing health system challenges due to complex patient profiles, patient expectations, and expanded demands [1,8].

Medication-related problems, including nonadherence, drug interactions, and adverse events, are gradually increasing in primary care [9,10,11,12,13]. In 2016–2017, it was reported that 250,000 hospital admissions and 400,000 presentations to emergency departments in Australia annually result from medication-related problems, and half of these hospital presentations are considered preventable [14,15]. Unplanned hospital admissions due to medication misadventure were estimated to have cost $1.4 billion to the Australian health care system in 2016–2017 [15].

Medication-related problems can occur due to poor communication and collaboration between community pharmacists and general practitioners [16]. The main barriers to communication include separate locations, limited face-to-face communication, and lack of routine access to patients’ medical records [17]. The inclusion of pharmacists in general practice teams is likely to address these barriers to collaboration and communication between pharmacists and general practitioners [18], and consequently may reduce adverse medication outcomes in primary care [19]. Pharmacists in general practice can identify and prevent medication-related problems [20], support general practitioners to improve the quality use of medicines, minimise the risks associated with medications, and improve patients’ health outcomes [19].

The integration of pharmacists into general practices has been studied in several countries, including Canada, United States of America (USA), United Kingdom (UK), Australia, Netherlands, and Ireland [19,20,21,22,23,24,25]. The National Health Service England (NHSE) launched the Clinical Pharmacists in General Practice model in 2015 to address the general practitioner workforce crisis [23]. Clinical pharmacists are employed to work as part of the general practice team to improve patient care and consult with patients directly. This includes providing extra help to manage chronic health conditions, education to those on multiple medicines, and better access to health assessments [26]. There are currently over 1000 full-time equivalent general practice pharmacists working across the UK, with a 38% increase of pharmacists in general practices year-on-year [27].

A large-scale project titled “Integrating Family Medicine and Pharmacy to Advance Primary Care Therapeutics (IMPACT)” has investigated the integration of pharmacists into family health teams since 2004–2006 in Ontario, Canada [24]. The IMPACT project aimed to improve collaborative practice between pharmacists, family physicians, and allied health professionals in managing medication therapy [24]. In this project, non-dispensing pharmacists provided patient medication interviews and assessments, medication management enhancement, patient education, and drug information [24,28]. Studies from Canada have reported the positive patient-centred activities of pharmacists as a part of the primary care team in providing integrated care [28,29].

In Australia, the pharmacist’s clinical role is generally limited to community pharmacies, hospitals, and consulting at aged-care facilities or when performing home medicines reviews [30]. Even though having clinical pharmacists working in general practice was first trialled in two projects in the early 2000s [31], this model has slowly expanded since around 2012. The concept of pharmacists working in general practice was supported by the Australian Medical Association, Pharmaceutical Society of Australia, and by various Primary Health Networks [32]. Limited number of pharmacists have been employed in general practice to provide various non-dispensing services, including patient education, medication reviews and other medication management, clinical audits, and administrative work [33]. Australian studies have indicated that integration of pharmacists into general practice is likely to reduce fragmentation of patient care and medication misadventure [34,35].

Interprofessional collaboration in healthcare is defined as multiple health professionals from diverse disciplines working together with patients, families, caregivers, and communities to deliver the highest quality of care [36]. A formal care team of health professionals is characterised by collaborative practice agreements between professionals, and responsibilities are distributed among the team members under such agreements [37]. According to the good pharmacy practice guidelines developed by the International Pharmaceutical Federation and the World Health Organisation, multidisciplinary collaboration among health-care professionals has been emphasized as a key factor to improve patient safety [38]. Further, guidelines suggested that pharmacists should collaborate with other health care professionals to improve health outcomes [38].

Interprofessional collaboration not only improves patient care and outcomes; it is required to improve job satisfaction, and reduce medication-related problems, inefficiencies, and health-care costs [20,39,40]. Where there is lack of collaboration and communication in health care delivery, poor patient outcomes, such as hospital re-admissions and medication-related problems, are common [41,42]. In addition, the absence of collaboration can cause ineffective teamwork, poor team management, and fragmented care [43,44]. However, successful collaborative care is not easy to achieve and can be affected by problems linked to uncertainties over authority, inadequate understanding of others’ tasks and liabilities, and professional boundary friction when delivering patient care [40].

Team effectiveness in health care is defined as a team’s capacity to achieve its goals and objectives to optimise patient care [45]. Team effectiveness is a combination of team processes, including shared understanding of goals, role specification, conflict resolution, information exchange, and leadership [46]. Team effectiveness is essential for patient safety when patients are looked after by more than one health professional; however, it can be affected by instability of the teams, the culture of the workplace, and inability to resolve conflicts [46,47,48].

The Australian general practice team commonly includes multiple health professionals. Coordination and collaboration between the team of professionals, including general practitioners, psychologists, dieticians, pathologists, physiotherapists, nurse practitioners, and nurses, is essential to deliver effective patient care [49,50]. Similarly, general practices in the UK and Canada provide integrated care for their patients: they include general practitioners/family physicians, nurses or nurse practitioners, and at least one other allied health professional such as dietician or psychologist [51,52]. They have included pharmacists into their teams to work directly with general practitioners/physicians, other health care professionals, and patients with the aim of optimizing patients’ medication regimens, health outcomes, and coordination of care [24,26]. The inclusion of pharmacists into general practice is relatively new to Australian general practices. Therefore, interprofessional collaboration and team-effectiveness within a large team of health professionals may be challenging when a practice pharmacist is being included into the established teams in general practices. Prior studies that investigated general practice pharmacists in Australia have reported stakeholders’ perspectives on aspects of collaboration using a qualitative methodology [18,53]. However, there has been a paucity of research exploring how interprofessional collaboration develops between the general practice pharmacist and other health professionals in general practice, while the collaboration between general practitioners and community pharmacists and nurses/nurse practitioners has been relatively well investigated [54,55].

The aim of this study is to evaluate interprofessional collaboration and team effectiveness after the inclusion of pharmacists into the general practice team. This cross-national comparative mixed-method study has been designed comprising two parts, Part A and B.

## 2. The Study

### 2.1. Study Instruments

In this study, two surveys (as described below) will be utilized to assess interprofessional collaboration and team effectiveness. Semi-structured interviews will be utilized to describe participants’ perception on how interprofessional collaboration developed among general practice team members.

#### 2.1.1. Collaborative Care Survey (CCS)

The CCS was developed to assess professional interactions, exchange characteristics (relationship initiation, trustworthiness, and role specification), and commitment to collaboration (Additional file S1). The survey is adapted from validated instruments that assessed community pharmacist-general practitioner relationships [37,56,57]. Survey questions were modified to investigate practice pharmacists’ relationships with general practitioners and allied health professionals. Questions in part 2 of CCS were formed by modifying the Frequency of Interprofessional Collaboration Instrument for Pharmacists (FICI-P), which is a 10-item unidimensional indicator that describes collaborative behaviour between community pharmacists and general practitioners in primary care [56]. The Pharmacist-Physician Collaborative Instrument (PPCI) was used to develop questions in part 3 of the CCS. The PPCI is a validated survey tool to identify physician-community pharmacist collaboration [57]. The PPCI includes seven themes covering collaborative working relationships: collaborative care, commitment, dependence symmetry, bidirectional communication, trust, initiating behaviour, and conflict resolution [56]. Professional interaction is measured on a 4-point scale, while exchange characteristics and commitment to collaboration are measured on 5-point Likert scales, indicating 1 (strongly disagree) to 5 (strongly agree).

The face validity of the CCS was reviewed by experts including one psychologist/academic and nine pharmacists (UK = 3, Canada = 2, Australia = 4). Following the review by experts, several alterations were made to the questionnaire: for instance, by refining the clarity of contextual characteristics or amending the wording of Likert scale questions. Then questionnaires were modified by the researchers according to the recommendations made by the experts. The revised questionnaire (Additional file S1) was approved by the team of experts.

#### 2.1.2. Team Effectiveness Survey (TES)

Questions to assess team effectiveness were adapted by the research team from a previously validated 31-item “Primary care team dynamics” survey [58]. The TES is a 24-item questionnaire that covers team processes and team performance domains: team effectiveness, shared understanding, processes for accountability, conflict resolution, acting and feeling like a team, and perceived team effectiveness (Additional file S2). The respondents will be asked to indicate the extent of agreement or disagreement for the statements on a 1 to 5-point Likert scale. The TES has similarities with CCS when assessing team processes (such as shared understanding, role specification, and team cohesion) while it differs from the CCS when assessing team performance domains (such as team effectiveness and perceived team effectiveness).

Following a pre-test of TES with experts and respondents (*n* = 8), the wording was amended according to their recommendations (Additional file S2). The final questionnaire was approved by the research team.

#### 2.1.3. Semi-Structured Interviews

Semi-structured interviews will provide value in understanding the views of individuals on three main domains: role clarity, professional interaction (relationship, trust, communication, decision making), and collaboration (overall view, impact on team effectiveness, barriers, and facilitators) (Additional file S3). The interviews will gain participants’ in-depth perceptions on how interprofessional collaboration developed among team members during the study period. The semi-structured interview guide was pre-tested with an experienced general practice pharmacist. The interview guide (Additional file S3: Domain 2) was amended based upon the comments.

### 2.2. Part A—Interprofessional Collaboration and Team Effectiveness of General Practice Team in the Australian Capital Territory (ACT)

#### 2.2.1. Aims

This study aims to assess (i) changes in interprofessional collaboration over time, (ii) the facilitators/barriers for developing interprofessional collaboration, and (iii) the level of team effectiveness in general practice teams following the inclusion of pharmacists into general practice in the ACT.

#### 2.2.2. Study Design

This study comprises a multiphase sequential explanatory mixed-method design (QUAN-QUAL-QUAN), including surveys (collaborative care and team effectiveness surveys) and semi-structured interviews [59,60,61].

#### 2.2.3. Setting

This study will be conducted in eight general practices in the ACT, Australia where the general practice pharmacist model is being trialled in a program conducted by the Capital Health Network (CHN), the ACT’s primary health network [62].

#### 2.2.4. Intervention

Pharmacists will be employed in eight general practices in the ACT on a part-time basis (15 h per week) for 18 months to provide non-dispensing services according to their own skillset and the local workplace needs determined by each practice. General practice pharmacists’ non-dispensing services include providing patient education, medication reviews and other medication management services, medication safety initiatives such as clinical audits, and medication information to general practitioners. Following the inclusion of pharmacists into general practice teams, pharmacists will work with general practitioners and other health professionals to provide integrated care in these general practices.

#### 2.2.5. Participants and Recruitment

The general practices eligible for the study will be identified and recruited by the CHN. These practices will be selected to reflect government-subsidised and private billing, and various locations in the ACT. General practice pharmacists will be recruited by the eligible general practices considering the local workplace needs determined by each practice.

General practice pharmacists, general practitioners, and other health professionals (may include nurses, nurse practitioners, psychologists, dieticians, and physiotherapists) (*n* ≈ 113) from eight general practices in the ACT, Australia will be invited to participate in surveys. For the qualitative component, all the general practice pharmacists (*n* = 8) and a purposeful sample of general practitioners and other allied health professionals in general practices (*n* ≈ 16) will be recruited [63,64]. Purposive sampling will be continued until the sample requirements are met by recruiting at least one pharmacist, one general practitioner, and one other health professional from each general practice. If it is believed that theoretical saturation is reached prior to full recruitment, then recruitment will discontinue [65,66].

#### 2.2.6. Inclusion and Exclusion Criteria

All potential participants who provide informed consent will be included in the study.

#### 2.2.7. Data Collection

The CCS and TES will be administered to general practice pharmacists electronically (Qualtrics, Provo, UT). General practitioners and other health professionals will be provided with paper-based surveys. All the participants will be invited to complete the CCS at two time points, at baseline and 12 months [23]. The TES responses will be gathered only once from all the participants, at 12 months. The surveys will be opened for 8 weeks. The summary of the study sequence is shown in Table 1.

Qualitative data will be collected through semi-structured interviews, which will be conducted by one of the experienced researchers (L.S.D.) via telephone after obtaining the written consent at 12 months. Full interviews will be audiotaped, de-identified, and transcribed verbatim by an independent professional transcribing service [63,67]. Field notes will be used to supplement the audio and transcripts.

#### 2.2.8. Data Analysis

Descriptive statistics will be used to summarise the demographic details of the participants. Reliability analysis (Cronbach’s alpha test) will be conducted to assess the internal consistency of multiple items of CCS and TES [68].

Total CCS scores will be calculated for general practice pharmacists, general practitioners, and other health professionals [69]. Total scores for the following CCS elements will be computed at the baseline and 12 months: professional interactions (range 1–4), relationship initiation (range 1–5), exchange characteristics (role specification and trustworthiness; range 1–5), and commitment to collaboration (range 1–5). The Kruskal–Wallis test, Wilcoxon-signed rank test, or Analysis of Variance (ANOVA) test, as appropriate, will be used to assess the differences in CCS variables within and between different participant groups at baseline and 12 months [70,71]. Total scores for TES will be calculated for the participant groups. The differences in TES scores between participant groups will be assessed using Kruskal–Wallis or ANOVA test as appropriate [58,70]. The data will be analysed using Statistical Package for the Social Sciences (SPSS ver. 25 IBM, New York, NY, USA).

Interview data will be coded and analysed independently by two investigators. Thematic analysis will be performed to analyse qualitative data [72]. The qualitative data analysis will utilise open and axial coding of transcripts [73]. The emerging themes will be reviewed by the research team with the aim of using the themes to explain the factors that describe interprofessional collaboration. Following the initial coding, a process of selective coding will be undertaken [63,67]. Thematic analysis will be performed with the assistance of NVivo qualitative data analysis software (ver. 12, QSR, Melbourne, VIC, Australia) [72]. Quantitative and qualitative results will be merged during interpretation to provide complementary perspectives of interprofessional collaboration.

#### 2.2.9. Outcomes

The primary outcomes of this study are the level of team effectiveness and the changes in interprofessional collaboration over time, after a general practice pharmacist commences employment in general practices in the ACT. Using a longitudinal comparison between baseline and 12 months after including a pharmacist in general practice, the changes in professional interactions, exchange characteristics (relationship initiation, trustworthiness, and role specification) and commitment to collaboration will be identified. Competencies for interprofessional collaboration and facilitating and impeding factors for collaborative care will be identified by conducting semi-structured interviews.

### 2.3. Part B—Interprofessional Collaboration and Team Effectiveness of General Practice Pharmacists in Australia, Canada, and the UK

#### 2.3.1. Aims

Part B aims to compare the level of interprofessional collaboration and team effectiveness in general practices in Australia with international sites (Canada and the UK), where the model of pharmacists working in general practice is more established [28,30].

#### 2.3.2. Study Design

A cross-national quantitative descriptive design (CCS and TES at one time point) will be utilised to compare the level of interprofessional collaboration and team effectiveness between Australia and Canada and the UK; and explore the demographic and contextual factors that influence interprofessional collaboration and team effectiveness of general practice pharmacists in Australia compared to Canada and the UK.

#### 2.3.3. Setting

This study will be conducted in general practices in Australia where the inclusion of pharmacists into general practices has only recently expanded, and general practices in two countries, Canada and the UK, where the pharmacist in general practice model is relatively well developed [28,30]. General practices in Australia, Canada, and the UK include multiple health professionals to provide integrated care for patients.

#### 2.3.4. Participants and Recruitment

A sample of general practice pharmacists from Australian states/territories, Canada, and the UK will be invited to participate in the online surveys. When calculating the sample size for a representative sample with a 95% confidence interval and 5% margin of error, 63 responses from Australia (total general practice pharmacists *n* ≈ 75 [74], 323 responses from the UK (total general practice pharmacists *n* ≈ 2000) [27] and 249 from Canada (total family practice pharmacists *n* ≈ 700 [75]) will be required to adequately reflect the entire population of general practice pharmacists from Australia and the international sites [76]. Potential participants from Australian states/territories and the international sites will be identified through professional networks and contacts in relevant professional organisations already established by the investigators. The surveys will be advertised in newsletters and through networks of professional organisations.

#### 2.3.5. Intervention

General practice pharmacists are working together with general practitioners and other health professionals to provide integrated patient care as routine practice. This study does not include any additional intervention.

#### 2.3.6. Inclusion and Exclusion Criteria

General practice pharmacists who are currently working in general practices in Australia, Canada, and the UK will be offered to participate in this study. General practice pharmacists described in Section 2.2.5 (i.e., involved in the ACT study) will be excluded from this study.

#### 2.3.7. Data Collection

Quantitative data will be collected from general practice pharmacists in Australia and international sites through the CCS and TES online (Qualtrics, Provo, UT, USA) at one time point. These surveys will be available for six months for the participants.

#### 2.3.8. Data Analysis

Descriptive statistics will be performed to summarise the demographic details of the participants. Total CCS and TES scores will be calculated for general practice pharmacists in Australia, Canada, and the UK. Spearman’s or Pearson’s correlation test will be performed to assess the relationship of professional interactions, relationship initiation, role specification, trustworthiness, team effectiveness, context characteristics, and individual characteristics, as appropriate. The Kruskal–Wallis or ANOVA test, as appropriate, will be performed to assess the differences in CCS variables between different countries [70]. Multiple linear regression will be performed to determine whether contextual and individual characteristics influence the total survey scores [37]. Collinearity will be assessed by computing the variance inflation factor.

#### 2.3.9. Outcomes

In Part B of the study, the primary outcome will be the level of interprofessional collaboration and team effectiveness across general practices in Australia, where the practice pharmacist model is relatively new, compared to general practices in the UK and Canada, where the practice pharmacist model is more established. The secondary outcome will be identifying the factors that influence interprofessional collaboration in three different sites. The findings from this study will offer insight into the primary aspects of strengthening interprofessional collaboration and team effectiveness following the inclusion of pharmacists into general practice teams in Australia.

### 2.4. Quality Control Measures

Quality control measures will be followed in carrying-out research procedures and handling data. Microsoft Excel spreadsheets will be used for data entry and organisation. Data entries will be verified by two investigators to ensure the accuracy and to minimise errors when processing data [77]. If the cases with missing data are more than 5%, multiple imputation will be performed to maximise the use of available information; and if the cases with missing data are less than 5%, listwise deletion will be performed to minimise bias [78,79].

## 3. Ethics

Ethical approval was obtained from the Human Research Ethics Committee of the University of Canberra (study protocol approval HREC 15-235).

### Data Management and Protection

Anonymous data collected from health professionals in general practices will be stored electronically on a secure password-protected hard drive and transferred to a secure password-protected server. Access to the database will be by the key members of the research team with unique usernames and passwords. The servers are protected by firewalls and are maintained according to best practice. Hard copies of data (i.e., completed questionnaires and consent forms) will be stored in a secured filing cabinet in the university. Audio-recordings will be transcribed and stored electronically, and the original recording will be deleted; all transcripts will be completely anonymised. After the completion of the study and publication of the results, the data will remain on the university storage system for five years, as per National Health and Medical Research Council (NHMRC) guidelines [80].

## 4. Discussion

This study will explore the extent and factors that influence collaboration and team effectiveness in general practice teams following the introduction of pharmacists into general practices in Australia. There is limited quantitative data regarding interprofessional collaboration in general practices worldwide [81]. Gaining insights related to interprofessional collaboration and team effectiveness will be an essential step to enhance general practice pharmacists’ services in Australia.

Furthermore, collaborative care and team effectiveness measures will be compared with comparator sites in Canada and the UK, where this practice model is more established [28,30]. The characteristics of the models of general practice pharmacists vary in these three sites. In the UK, the NHS has funded the inclusion of pharmacists in general practices, predominantly to reduce the general practitioners’ workload and offer patients greater access to health services [26]. Furthermore, in the UK, general practice-based pharmacists have been offered independent and supplementary prescribing rights [82]. In Canada, the main focus of integrating pharmacists into primary care teams was to improve medication management through collaborative practice [24,29]. Like the UK, Canadian pharmacists in primary care teams can prescribe in some states/provinces [83].

In Australia, pharmacists are gradually being included in general practices to optimize patient health outcomes and support general practitioners to minimise medication-related problems [19]. The UK and Canada have been much more pro-active in the inclusion of pharmacists in patient care teams than in Australia. Australia lags behind in the inclusion of pharmacists into general practices due to health policy challenges and a lack of funding [64,84]. The functions and aims of general practice models in Australia, Canada, and the UK have shown similarities; however, regulations, remuneration, and implementation appear to be different [82,83,84].

### 4.1. Strengths and Limitations

A multiphase sequential explanatory mixed-method approach is the most suitable method to merge quantitative and qualitative findings during interpretation to provide complementary perspectives on inter-related contextual factors of interprofessional collaboration [57,85]. A mixed-methods approach prevents intrinsic biases that can arise in single method, single observer, and single theory studies [58,86]. Qualitative data analysis of the study will be performed by two researchers independently; this process ensures transparency in data coordination and interpretation [87]. The survey tools, which have been validated through previous studies, were modified for this study to apply to the general practice setting and pre-tested before being utilised in the study. Additionally, to improve the generalizability of findings, this study will compare the results across international sites as comparators where this integrated health care model is well established [28,30,88]. To our knowledge, this will be the first study to capture interprofessional collaboration and team effectiveness of general practice pharmacists in Australia compared to other countries.

There are limitations to the proposed study design. In Part A of the study, the small sample size and predetermined locations with the constrained availability of general practices employing a pharmacist are identified as limitations. However, a similar sample size has been utilised in prior studies to explore the general practice pharmacist model [63,64,89]. In the ACT, the recruited general practices may be more receptive to the inclusion of a general practice pharmacist into their team, and this may increase the risk of bias.

### 4.2. Implications

The quantitative and qualitative data obtained from this study will contribute to the paucity of literature in this area. Stakeholders and policymakers will benefit from the findings of the study through any recommendations to strengthen interprofessional collaboration and team effectiveness when integrating pharmacists into Australian general practices.

### 4.3. Study Status

To date, eight general practices in the ACT have been recruited by the CHN. All the general practices had recruited general practice pharmacists by March 2020. Quantitative and qualitative data collection has commenced in the ACT; quantitative data collection has commenced in Australia, Canada, and the UK.

## Figures and Tables

**Table 1:** Summary of the study sequence.

	Part A	Part B
**Design**	Multiphase sequential explanatory mixed method	Cross-national quantitative descriptive
**Setting**	Eight general practices in the ACT where a program nested with CHN	General practices in Australia, Canada, and the UK
**Participants**	General practice pharmacists, general practitioners and other health professionals (may include nurses, nurse practitioners, psychologists, dieticians, and physiotherapists)	General practice pharmacists
**Sample size**	Pharmacists, GPs and other health professionals from 8 general practices in the ACT; target sample size *n* = 113 for the surveys, and *n* = 24 for the interviews	All general practice pharmacists; target sample size *n* = 323 from the UK, *n* = 249 from Canada, *n* = 63 from Australia
**Inclusion and exclusion criteria**	Inclusion: Participants who provide informed consent	Inclusion: General practice pharmacists who are currently working in general practices in Australia, Canada, and the UK. Exclusion: General practice pharmacists in Part A of the study
**Intervention**	Part-time pharmacists (15 hrs/week) will be employed into 8 general practices for 18 months to provide non-dispensing services such as providing patient education, medication management services, medication safety initiatives such as clinical audits, and medication information to general practitioners.	The general practice pharmacist model is already established as a routine practice
**Data collection**	Collaborative care survey at baseline and 12 months, Team effectiveness survey at 12 months, and semi-structured interviews at 12 months	Collaborative care survey and Team effectiveness survey
**Outcomes**	Changes in collaboration and team effectiveness, and identify barriers and facilitators of interprofessional collaboration after including general practice pharmacists	Identify the level of interprofessional collaboration and team effectiveness, and the factors associated with collaboration at different study sites

CHN: Capital Health Network, ACT’s primary health network, GP: General practitioner.

## Data Availability

Not applicable.

## Supplementary Materials

The following are available online at https://www.mdpi.com/1660-4601/18/3/966/s1, Additional file S1—CCS to assess interprofessional collaboration, Additional file S2—Team effectiveness survey, Additional file S3—Semi-structured interview guide
