# Supplementary material for: Evaluation of General Practice Pharmacists: Study Protocol to Assess Interprofessional Collaboration and Team Effectiveness
Source: Int J Environ Res Public Health. 2021 Jan 22;18(3):966. doi: 10.3390/ijerph18030966 (PMC7908139; doi:10.3390/ijerph18030966)
Supplement: Supplementary file 1 [file ijerph-18-00966-s001.pdf]

Additional file S1- CCS to assess interprofessional collaboration

Collaborative care and team effectiveness survey for general practitioners (ACT)

Part 1 - Demographic details

1. What is your age (Years)?

☐ 20 - 30 ☐ 31 – 40 ☐ 41 – 50 ☐ 51 – 60 ☐ > 60

2. What is your gender?

☐ Male ☐ Female ☐ Other ☐ Prefer not to say

3. How many years of experience do you have?

☐ Less than 5 ☐ 5 – 11 ☐ 12 - 18 ☐ 19 – 25 ☐ More than 25

4. Which best describes your employment status in general practice?

☐ Part-time ☐ Full-time

5. How frequently do you contact the pharmacist in general practice?

☐ Never ☐ 1-2 times/week ☐ 3-4 times/week ☐ 5 times or more/week

6. What is the most frequent method of communication between you and the pharmacist in general practice? (Select all the options that apply to you)

☐ Face to face ☐ Telephone ☐ Electronic

☐ Other (please specify) ____

7. How long have you been working with the pharmacist in general practice?

☐ Less than 6 months ☐ 6 - 12 months

☐ 12 - 24 months ☐ More than 24 months

Part 2 - Professional interactions

How frequently did the following activities occur in the LAST MONTH?

I contacted the pharmacist to discuss a patient's drug-related problem(s)

☐ Nil ☐ 1 - 2 times ☐ 3-4 times ☐ 5 times or more

The pharmacist informed me about new products/services available

☐ Nil ☐ 1 - 2 times ☐ 3-4 times ☐ 5 times or more

I contacted the pharmacist for drug information

☐ Nil ☐ 1 - 2 times ☐ 3-4 times ☐ 5 times or more

The pharmacist contacted me to discuss a patient's drug-related problem(s)

☐ Nil ☐ 1 - 2 times ☐ 3-4 times ☐ 5 times or more

I referred a patient to the pharmacist

☐ Nil ☐ 1 - 2 times ☐ 3-4 times ☐ 5 times or more

Part 3 - Exchange characteristics (Relationship initiation, role specification, trustworthiness)

Please select the answer that applies to you.

I spent time trying to learn how I can help the pharmacist to provide better care

☐ Not at all ☐ To a less extent ☐ To some extent ☐ To a moderate extent ☐ To a great extent ☐ No opinion/ Not applicable

I provided information to the pharmacist about a specific patient

☐ Not at all ☐ To a less extent ☐ To some extent ☐ To a moderate extent ☐ To a great extent ☐ No opinion/ Not applicable

I showed an interest in helping the pharmacist to improve his/her practice

☐ Not at all ☐ To a less extent ☐ To some extent ☐ To a moderate extent ☐ To a great extent ☐ No opinion/ Not applicable

Please select the answer that applies to you. *(NA- "Not applicable")*

The pharmacist is a credible professional

☐ Strongly disagree ☐ Disagree ☐ Neutral ☐ Agree ☐ Strongly agree ☐ N/A

My interactions with the pharmacist are characterised by open communication of both parties

☐ Strongly disagree ☐ Disagree ☐ Neutral ☐ Agree ☐ Strongly agree ☐ N/A

I count on the pharmacist to do what he/she says

☐ Strongly disagree ☐ Disagree ☐ Neutral ☐ Agree ☐ Strongly agree ☐ N/A

I intend to keep working together with the pharmacist

☐ Strongly disagree ☐ Disagree ☐ Neutral ☐ Agree ☐ Strongly agree ☐ N/A

I trust the pharmacist's knowledge on drugs

☐ Strongly disagree ☐ Disagree ☐ Neutral ☐ Agree ☐ Strongly agree ☐ N/A

Communication between the pharmacist and I should be two-way

☐ Strongly disagree ☐ Disagree ☐ Neutral ☐ Agree ☐ Strongly agree ☐ N/A

The pharmacist should depend on me as much as I depend on him/her

☐ Strongly disagree ☐ Disagree ☐ Neutral ☐ Agree ☐ Strongly agree ☐ N/A

I work with the pharmacist to overcome disagreements on his/her role in managing drug therapy

☐ Strongly disagree ☐ Disagree ☐ Neutral ☐ Agree ☐ Strongly agree ☐ N/A

The pharmacist and I should be mutually dependent on each other in caring for patients

☐ Strongly disagree ☐ Disagree ☐ Neutral ☐ Agree ☐ Strongly agree ☐ N/A

The pharmacist and I should negotiate to come to an agreement on the activities in managing drug therapy

☐ Strongly disagree ☐ Disagree ☐ Neutral ☐ Agree ☐ Strongly agree ☐ N/A

Part 4 – Commitment to collaboration

Please select the answer that applies to you. *(N/A- “Not applicable”)*

There is cooperation between the pharmacist and my-self in managing drug therapy of our patients

☐ Strongly disagree ☐ Disagree ☐ Neutral ☐ Agree ☐ Strongly agree ☐ N/A

In making decisions for our patients, both mine and the pharmacist’s options are considered

☐ Strongly disagree ☐ Disagree ☐ Neutral ☐ Agree ☐ Strongly agree ☐ N/A

Decision making is coordinated between the pharmacist and me

☐ Strongly disagree ☐ Disagree ☐ Neutral ☐ Agree ☐ Strongly agree ☐ N/A

Decision making responsibilities for the patient’s drug therapy are shared between the pharmacist and myself only whenever necessary

☐ Strongly disagree ☐ Disagree ☐ Neutral ☐ Agree ☐ Strongly agree ☐ N/A

If you have any other comments or suggestions regarding collaborative care, please write in the provided space.

____

____

____

____

____

____

____

Thank you for your time and interest in completing the survey.

Collaborative care and team effectiveness survey for other healthcare professionals in general practice (ACT)

Part 1 - Demographic details

For each question below, write in the answer or select the answer applies to you.

1. What is your profession?

☐ Nurse practitioner ☐ Nurse ☐ Psychologist ☐ Dietitian

☐ Other (please specify) ____

2. What is your age (Years)?

☐ 20 - 30 ☐ 31 – 40 ☐ 41 – 50 ☐ 51 – 60 ☐ > 60

3. What is your gender?

☐ Male ☐ Female ☐ Other ☐ Prefer not to say

4. How many years of experience do you have?

☐ Less than 5 ☐ 5 – 11 ☐ 12 – 18 ☐ 19 – 25 ☐ More than 25

5. Which best describes your employment status in general practice?

☐ Part-time ☐ Full-time

6. How frequently do you contact the pharmacist in general practice?

☐ Never ☐ 1-2 times/week ☐ 3-4 times/week ☐ 5 times or more/week

7. What is the most frequent method of communication between you and the pharmacist in general practice? (Select all the options that apply to you)

☐ Face to face ☐ Telephone ☐ Electronic

☐ Other (please specify) ____

8. How long have you been working with the pharmacist in general practice?

☐ Less than 6 months ☐ 6 - 12 months

☐ 12 - 24 months ☐ More than 24 months

Part 2 - Professional interactions

How frequently did the following activities occur in the LAST MONTH?

I contacted the pharmacist to discuss a patient's drug-related problem(s)

☐ Nil ☐ 1 - 2 times ☐ 3-4 times ☐ 5 times or more

The pharmacist informed me about new products/services available

☐ Nil ☐ 1 - 2 times ☐ 3-4 times ☐ 5 times or more

I contacted the pharmacist for drug information

☐ Nil ☐ 1 - 2 times ☐ 3-4 times ☐ 5 times or more

The pharmacist contacted me to discuss a patient's drug-related problem(s)

☐ Nil ☐ 1 - 2 times ☐ 3-4 times ☐ 5 times or more

I referred a patient to the pharmacist

☐ Nil ☐ 1 - 2 times ☐ 3-4 times ☐ 5 times or more

Part 3 - Exchange characteristics (Relationship initiation, role specification, trustworthiness)

Please select the answer that applies to you.

I spent time trying to learn how I can help the pharmacist to provide better care

☐ Not at all ☐ To a less extent ☐ To some extent ☐ To a moderate extent ☐ To a great extent ☐ No opinion/ Not applicable

I provided information to the pharmacist about a specific patient

☐ Not at all ☐ To a less extent ☐ To some extent ☐ To a moderate extent ☐ To a great extent ☐ No opinion/ Not applicable

I showed an interest in helping the pharmacist to improve his/her practice

☐ Not at all ☐ To a less extent ☐ To some extent ☐ To a moderate extent ☐ To a great extent ☐ No opinion/ Not applicable

Please select the answer that applies to you. *(N/A- “Not applicable”)*

The pharmacist is a credible professional

☐ Strongly disagree ☐ Disagree ☐ Neutral ☐ Agree ☐ Strongly agree ☐ N/A

My interactions with the pharmacist are characterised by open communication of both parties

☐ Strongly disagree ☐ Disagree ☐ Neutral ☐ Agree ☐ Strongly agree ☐ N/A

I count on the pharmacist to do what he/she says

☐ Strongly disagree ☐ Disagree ☐ Neutral ☐ Agree ☐ Strongly agree ☐ N/A

I intend to keep working together with the pharmacist

☐ Strongly disagree ☐ Disagree ☐ Neutral ☐ Agree ☐ Strongly agree ☐ N/A

I trust the pharmacist’s knowledge on drugs

☐ Strongly disagree ☐ Disagree ☐ Neutral ☐ Agree ☐ Strongly agree ☐ N/A

Communication between the pharmacist and I should be two-way

☐ Strongly disagree ☐ Disagree ☐ Neutral ☐ Agree ☐ Strongly agree ☐ N/A

In providing patient care, I need the pharmacist as much as the pharmacist needs me

☐ Strongly disagree ☐ Disagree ☐ Neutral ☐ Agree ☐ Strongly agree ☐ N/A

The pharmacist and I mutually work with each other in caring for patients

☐ Strongly disagree ☐ Disagree ☐ Neutral ☐ Agree ☐ Strongly agree ☐ N/A

Part 4 – Commitment to collaboration

Please select the answer that applies to you. *(NA- "Not applicable")*

There is cooperation between the pharmacist and my-self in managing drug therapy of our patients when applicable

☐ Strongly disagree ☐ Disagree ☐ Neutral ☐ Agree ☐ Strongly agree ☐ N/A

There is a cooperation between the pharmacist and my-self in caring of our patients

☐ Strongly disagree ☐ Disagree ☐ Neutral ☐ Agree ☐ Strongly agree ☐ N/A

If you have any other comments or suggestions regarding collaborative care, please write in the provided space.

____

____

____

____

____

____

____

Thank you for your time and interest in completing the survey.

Collaborative care and team effectiveness survey for pharmacists in general practice/family practice

(Australia, UK, Canada)

Part 1 - Demographic details

For each question below, write in the answer or select the answer applies to you.

1 What is your age (Years)?

○ 20 – 30 ○ 31 – 40 ○ 41 – 50 ○ 51 – 60 ○> 60

2 What is your gender?

○ Male ○ Female ○ Other ○ Prefer not to say

3 How many years of experience do you have as a pharmacist?

○ Less than 5 ○ 5 – 11 ○ 12 - 18 ○ 19 – 25 ○ More than 25

4 What is your previous working background prior to commencing your career as a practice pharmacist?

○ Hospital-based pharmacy practice ○ Community pharmacy-based practice

○ Other (please specify) ____

5 On average, what proportion (in %) do you currently work in the following areas of pharmacy?

General practice	%
Community pharmacy	%
Hospital pharmacy	%
Industry	%
Academia/ research	%
Total	

6 What is your location of your general practice? (Please write the state/ territory and the suburb in the provided space)- this question is only for the pharmacists in Australia

____

7 What are your academic and professional qualifications?

____

____

8 Are you annotated as a prescriber? (This question is only included in the surveys for UK and Canada)

○ Yes - annotated as an independent prescriber

○ Yes - annotated as a supplementary prescriber

○ Yes - annotated as both an independent and supplementary prescriber

○ No

9 How many general practitioners (GPs) are there in your practice(s)?

○ 1 – 4 ○ 5 – 9 ○ More than 10

10 Of the GPs that you are working with, how many do you work with closely? _______________

11 How frequently do you contact the GP(s)?

○ Never ○ 1-2 times/week ○ 3-4 times/week ○ 5 times or more/week

12 What is the most frequent method of communication between you and the GP(s)? (Select all the options that apply for you)

○ Face to face ○ Telephone ○ Electronic

○ Other (please specify) ____

13 How long have you been working in general practice?

○ Less than 6 months ○ 6 - 12 months

○ 12 - 24 months ○ More than 24 months

Part 2 - Professional interaction

For each statement below, select the answer that applies to you.

12 How frequently did the following activities occur in the **LAST MONTH?**

	Nil	1 - 2 times	3-4 times	5 times or more
I contacted a GP to discuss a patient's medicine-related problem(s)	○	○	○	○
I informed a GP of new products/services available	○	○	○	○
I was contacted by a GP for medicine information	○	○	○	○
I was contacted by a GP to discuss a patient's medicine-related problem(s)	○	○	○	○
I received a referral from a GP (to review medicines/educate/assess adverse effects/provide more information of medicines)	○	○	○	○

13 How frequently did you receive referrals from the following personnel in the **LAST MONTH**?

	Nil	1 - 2 times	3 - 4 times	5 times or more
Patients	○	○	○	○
Nurse	○	○	○	○
Psychologist/ mental health service	○	○	○	○
Dietitian	○	○	○	○
Reception staff	○	○	○	○
Care home manager/ support staff	○	○	○	○
Community pharmacist	○	○	○	○
Hospital pharmacist	○	○	○	○
Other allied health professional e.g. - physiotherapist, optometrist	○	○	○	○
Community organisations/ providers	○	○	○	○

Part 3 - Exchange characteristics (Relationship initiation, role specification, trustworthiness)

Complete this part of the survey based on your overall experience of working together with GP(s) to improve patient care.

14 Please select the answer that applies to you. (N/A- Not Applicable)

	Not at all	To a less extent	To some extent	To a moderate extent	To a great extent	No opinion/ N/A
I spent time trying to learn how I can help a GP to provide better care	○	○	○	○	○	○
I provided information to a GP about a specific patient	○	○	○	○	○	○
I showed an interest in helping a GP to improve his/her practice	○	○	○	○	○	○

15 Please select the answer that applies to you. (N/A- Not Applicable)

	Strongly disagree	Disagree	Neutral	Agree	Strongly agree	N/A
A GP is a credible practitioner	○	○	○	○	○	○
My interactions with a GP are characterised by open communication of both parties	○	○	○	○	○	○
I count on GP(s) to do what he/she says	○	○	○	○	○	○
I intend to keep working together with GP(s)	○	○	○	○	○	○
I trust the GP's drug expertise	○	○	○	○	○	○
Communication between a GP and me should be two-way	○	○	○	○	○	○
In providing patient care, I need GP(s) as much as GP(s) need(s) me	○	○	○	○	○	○
I should work with GP(s) to overcome disagreements on my role in managing medicines	○	○	○	○	○	○
A GP and I should be mutually dependent on each other in caring for patients	○	○	○	○	○	○
A GP and I should negotiate to come to an agreement on my activities in managing medicines	○	○	○	○	○	○

Part 4 – Commitment to collaboration

Complete this part of the survey based on your overall experience of working together with GP(s) to improve patient care.

16 Please select the answer that applies to you. (N/A- Not Applicable)

	Strongly disagree	Disagree	Neutral	Agree	Strongly agree	N/A
There is cooperation between a GP and pharmacist in managing medicines of our patients	○	○	○	○	○	○
Both GP's and pharmacist's options are considered in making decisions for our patients	○	○	○	○	○	○
Decision making is coordinated between a GP and pharmacist	○	○	○	○	○	○
Decision making responsibilities for the patient's medicines are shared between a GP and pharmacist, whenever necessary	○	○	○	○	○	○

17 If you have any other comments or suggestions regarding collaborative care, please write in the provided space.

____

____

____

Thank you for your time and interest in completing the survey.

Additional file S2 - Team effectiveness survey

Team effectiveness in general practice (This survey will be combined with CCS at the time of distribution).

Please select the answer that applies to you. (N/A- Not Applicable)

1. Membership of my team changes so frequently that we don't really have a team

☐ Strongly disagree ☐ Disagree ☐ Neutral ☐ Agree ☐ Strongly agree ☐ N/A

2. My team has the right "mix" of members—a group of people who bring different clinical perspectives and experiences to the work

☐ Strongly disagree ☐ Disagree ☐ Neutral ☐ Agree ☐ Strongly agree ☐ N/A

3. It is clear to my team what behaviour is acceptable / not acceptable

☐ Strongly disagree ☐ Disagree ☐ Neutral ☐ Agree ☐ Strongly agree ☐ N/A

4. Our practice recognises and reinforces teams that perform well

☐ Strongly disagree ☐ Disagree ☐ Neutral ☐ Agree ☐ Strongly agree ☐ N/A

5. My team has goals that are clear, useful, and appropriate to my practice

☐ Strongly disagree ☐ Disagree ☐ Neutral ☐ Agree ☐ Strongly agree ☐ N/A

6. There is a desire among team members to work collaboratively

☐ Strongly disagree ☐ Disagree ☐ Neutral ☐ Agree ☐ Strongly agree ☐ N/A

7. If asked, I could explain every team member's role and how they overlap

☐ Strongly disagree ☐ Disagree ☐ Neutral ☐ Agree ☐ Strongly agree ☐ N/A

8. My team encourages patients to be active participants in decisions about their care

☐ Strongly disagree ☐ Disagree ☐ Neutral ☐ Agree ☐ Strongly agree ☐ N/A

9. My team does a good job of helping patients understand their care plan

☐ Strongly disagree ☐ Disagree ☐ Neutral ☐ Agree ☐ Strongly agree ☐ N/A

10. The patient's needs and preferences are treated as an essential part of my team's decisions

☐ Strongly disagree ☐ Disagree ☐ Neutral ☐ Agree ☐ Strongly agree ☐ N/A

11. Each team member shares accountability for team decisions and outcomes

☐ Strongly disagree ☐ Disagree ☐ Neutral ☐ Agree ☐ Strongly agree ☐ N/A

12. My team has developed effective strategies for sharing patient treatment goals among team members

☐ Strongly disagree ☐ Disagree ☐ Neutral ☐ Agree ☐ Strongly agree ☐ N/A

13. Relevant information about changes in patient status or care plan is reported to the appropriate team member in a timely manner

☐ Strongly disagree ☐ Disagree ☐ Neutral ☐ Agree ☐ Strongly agree ☐ N/A

14. All team members effectively use the patient health record as a communication tool

☐ Strongly disagree ☐ Disagree ☐ Neutral ☐ Agree ☐ Strongly agree ☐ N/A

15. My team addresses patients' concerns effectively through team meetings and discussions

☐ Strongly disagree ☐ Disagree ☐ Neutral ☐ Agree ☐ Strongly agree ☐ N/A

16. Team meetings provide an open, comfortable, safe place to discuss concerns

☐ Strongly disagree ☐ Disagree ☐ Neutral ☐ Agree ☐ Strongly agree ☐ N/A

17. My team has an effective process for conflict management

☐ Strongly disagree ☐ Disagree ☐ Neutral ☐ Agree ☐ Strongly agree ☐ N/A

18. Overall, members of our team do a very good job of coordinating their different patient-related jobs and activities

☐ Strongly disagree ☐ Disagree ☐ Neutral ☐ Agree ☐ Strongly agree ☐ N/A

19. Members of my team act upon the information I communicate to them

☐ Strongly disagree ☐ Disagree ☐ Neutral ☐ Agree ☐ Strongly agree ☐ N/A

20. The way my team members interact makes the delivery of care highly efficient

☐ Strongly disagree ☐ Disagree ☐ Neutral ☐ Agree ☐ Strongly agree ☐ N/A

21. The way my team members interact is very good for the quality of patient care

☐ Strongly disagree ☐ Disagree ☐ Neutral ☐ Agree ☐ Strongly agree ☐ N/A

22. Working on a team like mine keeps members of my team enthusiastic and interested in their jobs

☐ Strongly disagree ☐ Disagree ☐ Neutral ☐ Agree ☐ Strongly agree ☐ N/A

23. I feel integral to my team

☐ Strongly disagree ☐ Disagree ☐ Neutral ☐ Agree ☐ Strongly agree ☐ N/A

24. I experience excellent teamwork with the members of my team

☐ Strongly disagree ☐ Disagree ☐ Neutral ☐ Agree ☐ Strongly agree ☐ N/A

Additional file S3 - Semi-structured interview guide

- Introduction
- Permission to tape/ any questions
- Ice breaker – What is your profession? How long have you been working in this practice?

Domain	Participants	Question
Role clarity	Pharmacists	What are your roles within this practice?<br>Can you describe your role? What has been the most satisfying aspect of your role in general practice?<br>Describe any benefits to patients of your role?<br>Describe any benefits to other healthcare professionals?<br>What qualities do you need in your role (s)? Why?<br>What skills do you need in your role(s)? Why?<br>What CPD have you undertaken since working in general practice? Why?<br>What CPD would be useful? Why?<br>What other healthcare professionals work in your practice? What do you understand by their roles?
	GPs and other HCPs	What are your roles within this practice?<br>Can you describe your role?<br>Can you describe the practice pharmacist's role? Describe the effect that the general practice pharmacist has had on your workload? What do you think has been the effect of employing a practice pharmacist on the patients?<br>What qualities do you think practice pharmacists need in their role? Why?<br>What skills do you think practice pharmacists need in their role? Why?<br>What CPD would you suggest for practice pharmacists? Why?
Professional interactions and trust	Pharmacists	What are your experiences in working with other HCPs in the practice?<br>Who is identifying patient's medication-related problems within the practice? Can you describe examples of how a patient's medication-related problems are resolved within the practice?<br>Can you describe your working relationship with GPs? Can you give examples of how you have interacted with them? What initiatives or activities have you done to establish collaboration with GPs? What worked? What did not?<br>Can you describe your working relationship with other healthcare professionals? Can you give examples of how you have interacted with them? What initiatives or activities have you done to establish collaboration with other healthcare professionals? What worked? What did not?<br>How much do you trust the work, decisions, views of GPs and other healthcare professionals? Have you experienced a situation

		where you feel your work or recommendation wasn't trusted by GP or other healthcare professional? How did that feel to you? How do you build trust with GPs and other healthcare professionals?<br>Can you explain communication process between you and GPs within the practice? How often during a usual week do you communicate with GPs? Has it always been this frequent? What is the method of communication? Has this changed?<br>How often during a usual week do you communicate with other healthcare professionals? Has it always been this frequent? What is the mode of communication? Has this changed?<br>Do you think that open communication occurs within your practice? Can you give some examples to support your answer to the previous question? Do you find it easy to communicate with GPs? Are there any barriers to communicating with your team? How do other team members encourage you to contribute to patient management during discussions?
	GPs and other HCPs	What are your experiences in working with practice pharmacist?<br>Can you describe examples of how a patient's medication-related problems are identified and resolved within the practice?<br>Can you describe your working relationship with practice pharmacist? Can you give examples of how you have interacted with the practice pharmacist? What initiatives or activities have you done to establish collaboration with practice pharmacist? What worked? What did not? Has this changed over time?<br>How much do you trust the work, decisions and views of the practice pharmacist? Have you experienced a situation where you feel your work or recommendation wasn't trusted by the practice pharmacist? How did that feel to you? How do you build trust with the practice pharmacist?<br>Can you explain communication process among health care professionals within the practice? How often during a usual week do you communicate with practice pharmacist? Has it always been this frequent? What is the mode of communication?<br>Do you find it easy to communicate with the pharmacist? Are there any barriers to communicating with the pharmacist? How do you encourage the pharmacist to contribute to patient management during discussions?
Collaboration and team effectiveness	Pharmacists GPs and other HCPs	What do you understand by interprofessional collaboration?<br>What were your initial thoughts on working with a team in general practice? Have they changed?<br>What is your overall view on collaborative care in general practice?

	What do you think is the impact of collaborative care on team effectiveness?<br>What are the barriers for collaboration between the practice pharmacist and GPs or healthcare professionals in general practice?<br>What are your suggestions to improve collaborative care involving the practice pharmacist in general practice?

*GP-General practitioner, HCP-Health care professional

- Is there anything else that you would like to tell me about your experiences with collaboration with other team members within general practice?
- Closing remarks
